# Aging Mechanisms and Performance Degradation of XLPE Submarine Cable Insulation Under Marine Major Anion Effects

**DOI:** 10.3390/polym17182450

**Published:** 2025-09-10

**Authors:** Liang Zou, Zheng Liu, Zhiyun Han, Shoushui Han, Guochang Li, Qingsong Liu

**Affiliations:** 1 School of Electrical Engineering, Shandong University, Jinan 250061, China; zouliang@sdu.edu.cn (L.Z.); 202334730@mail.sdu.edu.cn (Z.L.); 202434730@mail.sdu.edu.cn (S.H.); 2 College of Automation and Electronic Engineering, Qingdao University of Science and Technology, Qingdao 266042, China; lgc@qust.edu.cn; 3 Electric Power Research Institute, CSG EHV Power Transmission Company, Guangzhou 510700, China; 13560200175@163.com

**Keywords:** cross-linked polyethylene, submarine cable, anion-induced degradation, synergistic mechanism, aging mechanism

## Abstract

When the outer sheath of submarine cables is damaged, the degradation of cross-linked polyethylene (XLPE) insulation by anions in seawater becomes a critical factor affecting cable service life. This study investigates 500 kV three-core XLPE insulation and systematically reveals the differential and synergistic degradation mechanisms of major seawater anions (Cl^−^, SO_4_^2−^, HCO_3_^−^). Accelerated aging tests at 90 °C were conducted using solution systems simulating both single-ion and composite environments, combined with electrical performance evaluation, Fourier transform infrared spectroscopy (FTIR), and scanning electron microscopy (SEM). Results show that seawater causes significantly greater deterioration of resistivity, breakdown strength, and molecular structure than any single-ion solution. Mechanistic analysis demonstrates that Cl^−^ induces nucleophilic substitution, SO_4_^2−^ promotes oxidative chain scission, and HCO_3_^−^ facilitates hydrolysis via pH regulation, while their coexistence produces nonlinear synergistic effects through oxidative reactions, electrochemical coupling, and ion transport. This work provides the first systematic comparison of individual and combined anion effects on XLPE, offering new mechanistic insights and quantitative evidence for understanding multi-ion degradation, with implications for insulation material design, protective strategies, and service life prediction of submarine cables.

## 1. Introduction

Global energy transition and rapid development of offshore wind farms have made submarine cables indispensable key infrastructure in modern power transmission systems [1,2]. As important links connecting land with marine renewable energy facilities, the deployment scale and technical requirements of submarine cables continue to increase, with global submarine cable markets expected to achieve significant growth over the next decade [3,4,5]. Cross-linked polyethylene (XLPE), due to its excellent electrical insulation properties, mechanical strength, and chemical stability, has become the preferred material for high-voltage submarine cable insulation layers [6]. However, complex and variable marine environmental conditions as well as various risk factors during construction and laying pose severe challenges to the long-term stable operation of submarine cables [7,8]. High-voltage submarine cables typically consist of several concentric layers, including a conductor, conductor screen, XLPE insulation, insulation screen, metallic sheath, bedding, steel armor, and a polymeric outer sheath. Each layer serves a specific function, with the XLPE insulation acting as the primary dielectric barrier that ensures electrical reliability. Damage or degradation at the outer sheath directly exposes the insulation to seawater, significantly increasing the risk of aging and failure.

The most serious threat faced by submarine cables during operation stems from insulation failure caused by outer sheath damage [9,10]. Statistical data indicate that mechanical damage caused by human activities accounts for over 90% of submarine cable failures, with anchor damage being the primary threat factor [11]. After outer sheath damage, seawater directly contacts the XLPE insulation layer, and numerous anions in seawater penetrate the material’s microstructure under high-temperature conditions and trigger complex chemical reactions, leading to degradation processes such as molecular chain breakage, oxidation reactions, and polar functional group formation [12,13]. More critically, different anions in seawater (Cl^−^, SO_4_^2−^, HCO_3_^−^, etc.) possess differential chemical activities and degradation characteristics, and their synergistic effects on XLPE insulation performance far exceed the superposition effects of single environmental factors [14]. Therefore, deeply revealing the degradation mechanisms of different anions and their synergistic action patterns holds important engineering value and scientific significance for ensuring the full-lifecycle reliability of submarine cables. On this basis, the central scientific problem of the present work is to clarify how the major anions in seawater individually affect the physicochemical degradation of XLPE insulation, and how their coexistence leads to nonlinear synergistic effects that cannot be predicted from single-ion behavior.

Research on aging mechanisms of XLPE insulation materials in existing work mainly concentrates on three aspects. In electrical performance evaluation, researchers have assessed the degree of material aging through macroscopic electrical parameters such as dielectric spectrum and breakdown strength. Adeniyi et al. [15] demonstrated that dielectric loss factor in the low-frequency region is particularly sensitive to aging state, providing a methodological reference for monitoring dielectric changes under different ionic environments. Hu et al. [16] developed aging prediction models incorporating dynamic thermal resistance, which highlights the importance of coupling environmental stress with thermal effects, a principle relevant to the present accelerated aging framework. In microscopic mechanism research, characterization techniques such as infrared spectroscopy and scanning electron microscopy have been applied to reveal molecular structural evolution. Zhang et al. [17] showed that changes in the carbonyl index are strongly correlated with oxidation reactions, illustrating the utility of spectroscopic indices as degradation markers, while in the present work the methylene index is further adopted to characterize multi-ion effects. Hosier et al. [18] analyzed water molecule diffusion in XLPE through molecular simulations, demonstrating how penetrant transport accelerates degradation, which conceptually relates to the synergistic ion transport discussed here. In degradation mechanism aspects, existing research mainly focuses on the effects of single environmental factors. Virtanen et al. [19] examined the role of cations in water tree aging, confirming the importance of ion type in electrochemical pathways though without addressing anion-specific effects. Jiang et al. [20] investigated copper armor degradation under induced current, providing evidence for coupled electrical and electrochemical processes relevant to complex marine environments. Taken together, these works highlight the role of ion-specific processes and coupled mechanisms, yet systematic comparison of different anions and elucidation of their synergistic degradation effects on XLPE insulation remain largely unexplored, which constitutes the central objective of the present study.

This study aims to systematically reveal the differential degradation mechanisms and synergistic action patterns of major anions in seawater (Cl^−^, SO_4_^2−^, HCO_3_^−^) on XLPE insulation materials. Through designed solution systems simulating typical marine environments and single anion compositions, using 90 °C high-temperature accelerated aging tests combined with multi-scale characterization methods including electrical performance testing (volume resistivity, dielectric constant, breakdown field strength), Fourier transform infrared spectroscopy (FTIR), and scanning electron microscopy (SEM), this research comprehensively analyzes the influence patterns of different anions on XLPE molecular structure and macroscopic properties. This study provides important theoretical basis and technical support for optimization design, protection strategy formulation, and service life prediction of submarine cable insulation materials.

## 2. Experimental Materials and Methods

### 2.1. Sample Preparation

**Figure 1:**
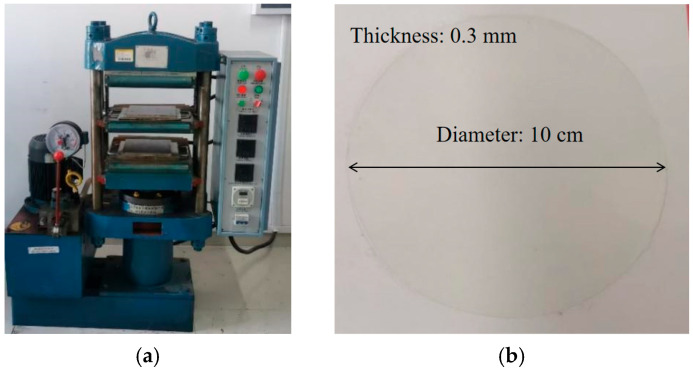
Preparation process and finished specimens. (**a**) Preparation process; (**b**) Finished sample.

Commercial 500 kV three-core cross-linked polyethylene (XLPE) cable insulation material was selected for the experiment. Prior to molding, the insulation material was dried in an oven to remove moisture. The material was then preheated without pressure at 130 °C for 4 min, followed by pressing at 130 °C for 6 min. Subsequently, a higher-temperature pressing stage was applied at 180 °C for 15 min to ensure complete crosslinking. Finally, the specimens were cooled under pressure at room temperature for 8 min. Insulation specimens with a uniform thickness of approximately 0.3 mm were thus obtained. The preparation process and finished specimens are shown in Figure 1.

### 2.2. Solution Preparation

Based on field surveys of the carbonate system in the South China Sea (SCS) [21], particularly on the northern shelf and adjacent reef/atoll regions, Cl^−^ and SO_4_^2−^ are consistently the dominant anions in seawater, while seawater alkalinity is overwhelmingly carried by HCO_3_^−^. Reported ranges for salinity and total alkalinity in the northern SCS are fully consistent with these ionic budgets. Therefore, the four solutions used in this work were formulated to match (i) single-anion endmembers for Cl^−^, SO_4_^2−^, and HCO_3_^−^ and (ii) a composite “seawater” mixture reflecting SCS field observations and open-ocean major-ion ratios; other anions are minor in concentration and were neglected [22,23,24]. Therefore, to investigate the corrosive effects of different anions and actual seawater environment on XLPE layers, four solutions were prepared according to published South China Sea research data: Solution No. 1 containing only Cl^−^, Solution No. 2 containing only SO_4_^2−^, Solution No. 3 containing only HCO_3_^−^, and Solution No. 4 simulating actual Qiongzhou Strait seawater by containing all three anions at concentrations matching those in the respective individual solutions.

Reagent amounts for the ionic concentrations were calculated and weighed using analytical balances. Dosages for preparing 1 L of each solution are detailed in Table 1. Chemicals were dissolved sequentially in about 800 mL pure water with stirring until complete, then transferred to a 1 L volumetric flask and diluted to the mark. Prepared solutions were stored in clean, sealed, date-labeled beakers to avoid contamination.

**Figure 2:**
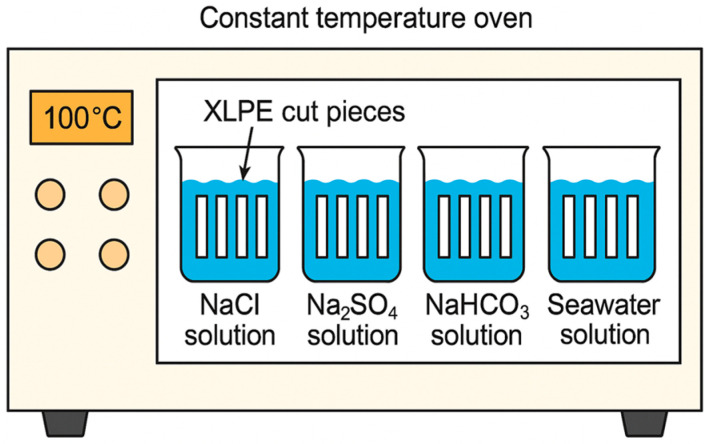
Aging test apparatus.

According to standard IEC 60216 [25], specimens were placed in thermal aging ovens with aging times set to 168 h, 336 h, 504 h, and 672 h, as shown in Figure 2. The long-term operating temperature of 500 kV cross-linked polyethylene cable insulation has been reported as approximately 90 °C [26], and therefore the target oven temperature was set to 100 °C. Prior to the experiments, the ovens were calibrated using a certified reference thermometer to ensure temperature accuracy and spatial uniformity within ±1 °C. During the tests, solution temperatures were continuously monitored with an independent thermometer, confirming a stable operating temperature of 90 °C with a maximum deviation of about 3 °C. To compensate for water loss caused by evaporation at elevated temperature, pure water was periodically added to each container to maintain constant solution volume and stable salt concentrations throughout the aging process.

### 2.3. Characterization Methods

A total of 16 specimens from solutions 1–4 at different aging times (4 specimens each) plus unaged specimens were subjected to the following tests. Prior to all measurements, specimens were rinsed with anhydrous ethanol and dried at 60 °C for 2 h to remove moisture, unless otherwise stated.

#### 2.3.1. Volume Resistivity Testing

**Figure 3:**
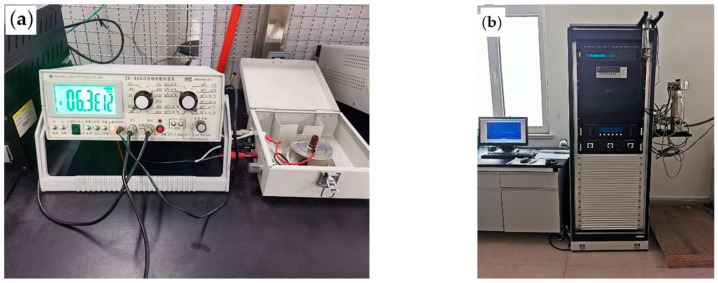
The testing process. (**a**) Volume resistivity measurement equipment; (**b**) Dielectric parameter measurement equipment.

Volume resistivity of specimens was measured using a ZC-90G High Insulation Resistance Meter at a test voltage of 1 kV. Each specimen was measured ten times, and the arithmetic mean was calculated. The resistivity measurement schematic is shown in Figure 3a. Volume resistivity was calculated using the following formula:

(1) $$\rho =R\frac{S}{h}$$

where ρ represents volume resistivity, *R* is the measured resistance value, *S* represents the contact area of the specimen, and *h* is the specimen thickness.

#### 2.3.2. Dielectric Property Testing

A Broadband Dielectric Spectrometer (Alpha-A, Novocontrol, Montabaur, Germany) was used to measure the dielectric constant and dielectric loss factor of XLPE specimens under different aging times. Before testing, metallic aluminum films were applied to specimen surfaces to ensure optimal contact between metal electrodes and specimen surfaces. The experimental frequency range was set from 10^−1^ Hz to 10^6^ Hz with a test voltage of 1 V. Circular specimens with a diameter of 20 mm and thickness of 0.3 mm were used for testing. The dielectric constant measurement schematic is shown in Figure 3b.

#### 2.3.3. Breakdown Field Strength Testing

This study employed AC Dielectric Breakdown Test System (DDJ-100 kV, Beijing Guance Jingdian Instrument Equipment Co., Ltd., Beijing, China) to conduct power frequency breakdown field strength tests on XLPE specimens under different solution environments and aging times. The testing process followed IEC 60243-1:2013 standard [27]. Applied voltage used AC 50 Hz power frequency with linear voltage rise at a rate of 500 V/s until specimen breakdown occurred, and breakdown voltage values were recorded. To avoid corona discharge phenomena, cylindrical electrodes with a diameter of 25 mm were selected for contact. Specimen thickness was uniformly 0.3 mm. The entire breakdown testing process was conducted in dimethyl silicone oil to prevent air breakdown interference and improve data stability and repeatability. For each test group, 10 independent tests were performed on specimens under identical aging conditions, and breakdown field strength data were statistically analyzed using two-parameter Weibull distribution. The breakdown probability distribution function is expressed as follows:

(2) $$F(t;\alpha ,\beta )=1-\beta \cdot exp(\frac{t}{\alpha })$$

where *t* represents experimentally obtained breakdown field strength data, *α* is the scale parameter (corresponding to breakdown field strength at 63.2% failure rate) reflecting specimen characteristic strength, and *β* is the shape parameter used to measure the dispersion degree of breakdown data. This distribution function effectively reflects the influence trends and statistical patterns of different corrosive environments and aging times on XLPE insulation performance.

#### 2.3.4. Fourier Transform Infrared Spectroscopy

FTIR spectra were obtained with a Nicolet™ iS50 Spectrometer (Thermo Fisher Scientific Inc., Waltham, MA, USA) to analyze the functional group variation patterns of XLPE specimens under different solutions and aging conditions. The scanning wavenumber range was set at 4000–500 cm^−1^.

#### 2.3.5. Scanning Electron Microscopy Testing

Scanning Electron Microscopy (SEM) was used with a Hitachi SU3500 Scanning Electron Microscope (Hitachi High-Technologies Corporation, Tokyo, Japan) to test surface degradation morphology of specimens under different solutions and aging times. Specimens removed from degradation apparatus were cleaned with anhydrous ethanol, followed by drying treatment to ensure no surface deposits remained. To improve SEM imaging clarity and conductivity, metallic films were coated on specimen surfaces. SEM was used to scan surface morphology of processed specimens with an acceleration voltage set at 15 kV, and scanning magnification was flexibly adjusted according to different degradation conditions.

## 3. Experimental Results and Analysis

### 3.1. Electrical Performance Variation Analysis

#### 3.1.1. Volume Resistivity Variation Pattern

**Figure 4:**
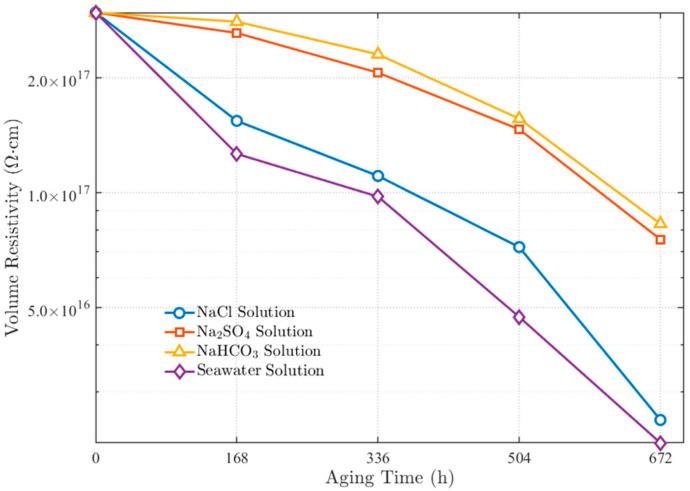
Variation patterns of XLPE volume resistivity with aging time in different anionic solutions.

The variation patterns of XLPE volume resistivity under different corrosive solutions are shown in Figure 4. A logarithmic scale was adopted for the vertical axis of resistivity to clearly present the wide range of values and highlight the relative differences among the various anionic environments.

For NaCl solution, the volume resistivity exhibited a pronounced declining trend with extended aging time, decreasing from the initial value of 2.97 × 10^17^ Ω·cm to 2.54 × 10^17^ Ω·cm after 672 h, representing a decrease of 91%. This change indicates that chloride ions possess strong chemical activity and can significantly accelerate the electrical performance degradation of XLPE material, resulting in substantial reduction of volume resistivity [28]. In Na_2_SO_4_ solution, although volume resistivity also showed a declining trend, the magnitude of decrease was smaller compared to NaCl solution, with a reduction of 74%. This suggests that while sulfate ions also cause degradation to XLPE material, their corrosive effect is weaker than that of chloride ions. For NaHCO_3_ solution, the volume resistivity decreased to 8.29 × 10^17^ Ω·cm after 672 h of aging, representing a decline of 72%. Although the variation trend was similar to that in Na_2_SO_4_ solution, the mechanism of bicarbonate ion action during aging differs from that of sulfate ions, as it promotes localized degradation and electrical performance degradation of the material by altering local pH values under high-temperature conditions. For the simulated seawater solution, the volume resistivity change was most significant, with a decline of up to 93% after 672 h of aging. This change reflects the synergistic effects of multiple anions in seawater, particularly the combined action of chloride ions with other anions, which greatly accelerates the electrical performance deterioration of XLPE material. The corrosive effect of seawater exceeds that of individual anionic solutions, especially under long-term exposure, where volume resistivity decreases rapidly.

The volume resistivity variation trends in different solutions reveal the differential corrosive effects of various anions on XLPE cables. Chloride ions exhibit the most significant corrosive effect on XLPE material, with their action intensity far exceeding that of sulfate and bicarbonate ions. Additionally, the synergistic effects of multiple anions in seawater further exacerbate the electrical performance deterioration of the material.

#### 3.1.2. Dielectric Performance Variation

**Figure 5:**
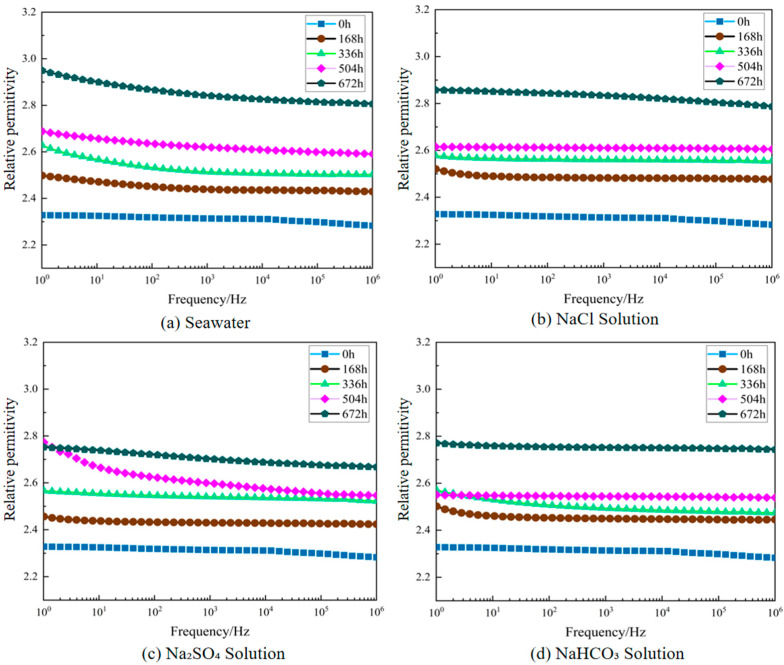
Changes in XLPE relative dielectric constant after degradation in different solutions. (**a**) Seawater solution; (**b**) NaCl solution; (**c**) Na_2_SO_4_ solution; (**d**) NaHCO_3_ solution.

**Figure 6:**
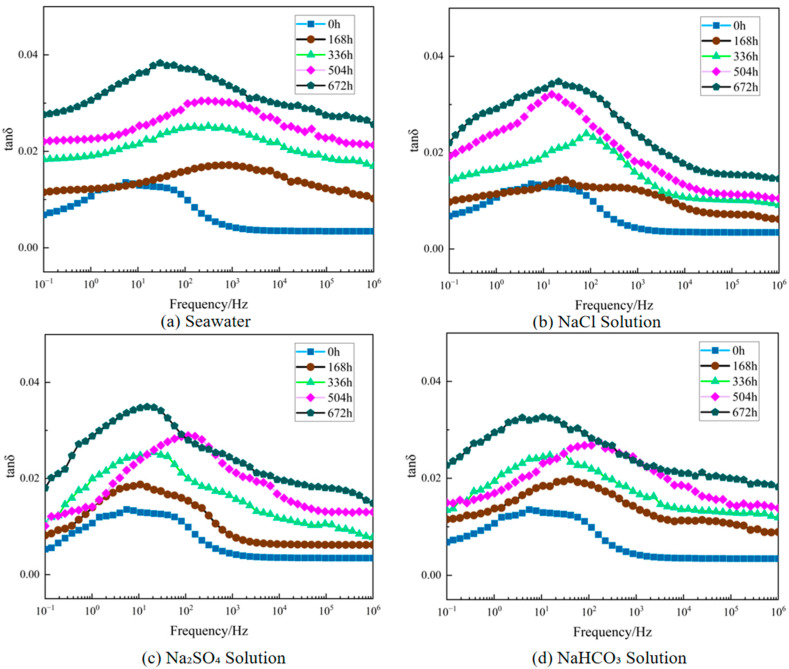
Changes in XLPE dielectric loss factor after degradation in different solutions. (**a**) Seawater solution; (**b**) NaCl solution; (**c**) Na_2_SO_4_ solution; (**d**) NaHCO_3_ solution.

**Figure 7:**
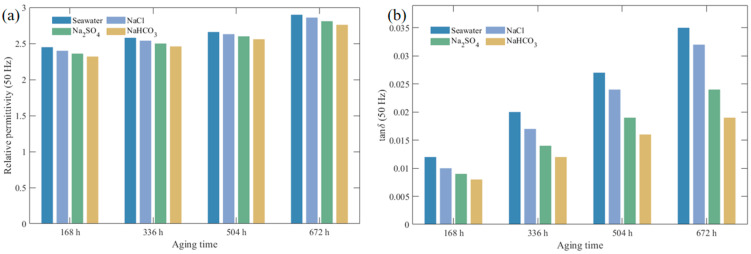
Relative dielectric properties of XLPE at 50 Hz after different aging times in different solutions. (**a**) Relative permittivity *ε*_r_; (**b**) Dielectric loss factor tan *δ*.

The variation patterns of XLPE dielectric constant at different aging time points in various corrosive solutions are shown in Figure 5, while the variation patterns of dielectric loss factor are shown in Figure 6 (the dielectric constants discussed in this paper are all relative values). To facilitate direct comparison among different solutions at power frequency, Figure 7 presents the relative permittivity and dielectric loss factor at 50 Hz in bar chart form.

Figure 5 and Figure 6 clearly demonstrate the typical frequency-dependent characteristics of dielectric performance for XLPE specimens under different corrosive media and aging times. Regardless of solution type and aging degree, both dielectric loss factor (tan *δ*) and relative dielectric constant (*ε*_r_) exhibited pronounced frequency dispersion behavior. In the high-frequency region, curves remained relatively stable with minimal changes in tan *δ* and *ε*_r_ values. At lower frequencies, both parameters increased markedly as frequency decreased, reflecting the frequency dependence of dielectric polarization processes [29]. A distinct tan *δ* peak can be observed in the low-frequency range, which becomes more evident with aging. This feature arises from the competition between polarization establishment and conduction processes: as frequency decreases, interfacial polarization and charge transport effects accumulate, leading to rising tan *δ* values until the peak frequency is reached. Beyond this point, relaxation of polarization mechanisms reduces dielectric losses, producing the subsequent decline. The presence and progressive shift of this peak under aging conditions indicate enhanced interfacial relaxation and increased density of polar degradation products and structural defects, consistent with reported dielectric loss behaviors in aged XLPE [30,31]. As shown in Figure 7, this comparison at 50 Hz clearly demonstrates that seawater causes the most severe deterioration, followed by NaCl, Na_2_SO_4_, and NaHCO_3_, consistent with the frequency-dependent trends observed in Figure 5 and Figure 6. Different corrosive media exhibited distinct effects on XLPE dielectric performance degradation. As shown in Figure 5 and Figure 6, under identical aging times, tan *δ* and *ε*_r_ degradation was most significant in seawater environment, exceeding that in individual salt solution environments. After 672 h of aging, the low-frequency tan *δ* of seawater-corroded specimens increased substantially compared to initial values and remained higher than specimens aged in NaCl, Na_2_SO_4_, or NaHCO_3_ solutions across the entire test frequency range. Chloride ions demonstrated particularly pronounced destructive effects on XLPE aging, strongly promoting polymer chain oxidation and moisture intrusion, significantly increasing dielectric constant and losses. In contrast, sulfate and bicarbonate ions exhibited relatively weaker corrosive effects: sulfate ions primarily induced general chemical degradation, while bicarbonate ions accelerated material hydrolysis at high temperatures by increasing local pH, but both showed less severe dielectric performance degradation compared to chloride ions when acting individually. Notably, the simultaneous presence of multiple anions in seawater exhibited synergistic degradation effects: the combined action of Cl^−^, SO_4_^2−^, HCO_3_^−^ and other ions accelerated the accumulation of polar degradation products and moisture penetration within XLPE, resulting in tan *δ* and *ε*_r_ increases in the low-frequency region far exceeding those in any single-ion environment.

#### 3.1.3. Breakdown Field Strength Degradation Characteristics

**Figure 8:**
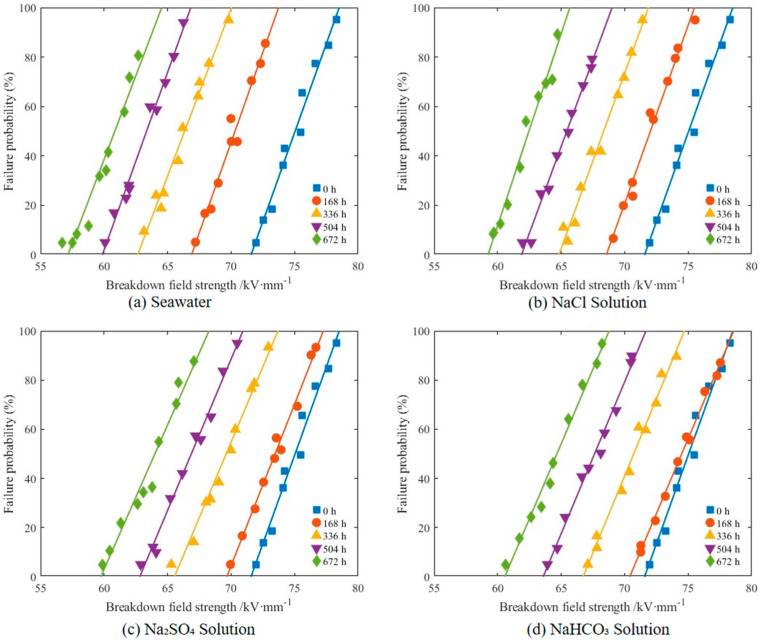
Weibull breakdown probability plots for different degradation times in different solutions. (**a**) Seawater solution; (**b**) NaCl solution; (**c**) Na_2_SO_4_ solution; (**d**) NaHCO_3_ solution.

The variation of XLPE insulation breakdown field strength with aging time in different corrosive solutions is shown in Figure 8, with corresponding Weibull distribution parameters listed in Table 2. Unaged XLPE specimens in all four solution environments exhibited identical breakdown characteristic field strength (α) values of 75.37 kV/mm and shape parameter (β) of 45.0. With increasing aging time, XLPE specimens in all solutions showed significant declining trends in breakdown field strength, but the magnitude and rate of decline varied depending on solution composition. After aging to 672 h, breakdown field strength decreased significantly in all four solutions, but with distinct degrees of degradation. In seawater solution, α decreased to 57.84 kV/mm, while in NaCl, Na_2_SO_4_, and NaHCO_3_ solutions, α values were 57.90 kV/mm, 60.65 kV/mm, and 64.00 kV/mm, respectively, representing decreases of 17.53, 17.47, 14.72, and 11.37 kV/mm, demonstrating that Cl^−^ exhibited the strongest corrosive effect, with multi-anion synergistic action further exacerbating breakdown performance degradation. In contrast, β value changes revealed the impact of various solutions on specimen failure consistency. In NaHCO_3_, β decreased from 45.0 to 27.5, in Na_2_SO_4_ to 26.3, while in NaCl and seawater, β decreased to 22.7 and 20.9, respectively, indicating that Cl^−^ and its synergistic degradation effects in composite electrolytes not only accelerated structural degradation but also enhanced the randomness of breakdown behavior. This declining trend in β values suggests that defect distribution within the material became increasingly non-uniform during aging, with structural integrity compromised to varying degrees.

With extended aging time, XLPE breakdown performance continued to deteriorate. The combined effects of thermal oxidation and chemical degradation damaged XLPE macromolecular chains, leading to partial destruction of crystalline regions and their transformation into amorphous regions within the material. Under electric field application, charge carriers primarily migrate along interfaces between crystalline and amorphous regions [32]. Damage to crystalline structure facilitates charge carrier migration, thereby reducing the dielectric breakdown resistance. During AC breakdown testing, when voltage is in the negative half-cycle, negative charges injected into the material are trapped by microscopic defect traps; during polarity reversal into the positive half-cycle, newly injected positive charges recombine with previously accumulated negative charges and release energy. As aging progresses, more shallow-level traps form within XLPE, continuously reducing the critical field strength required for charge injection, thereby increasing space charge injection and intensifying positive-negative charge recombination with greater energy release, ultimately resulting in significant breakdown field strength reduction in aged specimens. Although the measured breakdown voltages appear numerically similar among different solutions, analysis of Weibull distribution parameters provides further insight: variations in the scale parameter α reflect differences in degradation rates of breakdown strength, while changes in the shape parameter β indicate distinct levels of statistical consistency across environments. Multi-ion seawater conditions exhibited reduced α values and lower β values compared to single-ion cases, confirming that synergistic anion effects accelerate aging and induce greater variability in breakdown resistance. Particularly, the multi-ion environment in seawater accelerated XLPE oxidative degradation product accumulation and moisture penetration, further increasing trap density and charge carrier mobility, making breakdown strength reduction especially pronounced in this environment.

**Figure 9:**
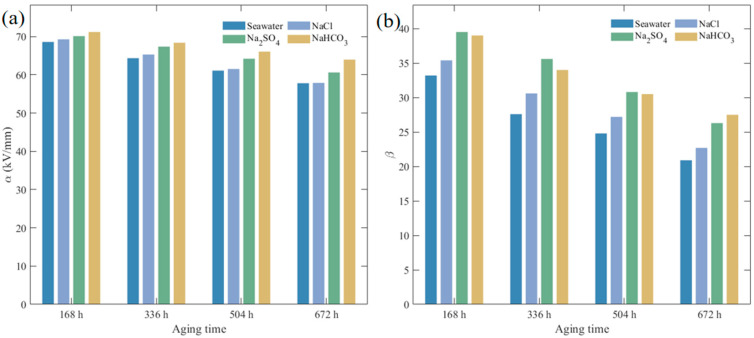
Variations of Weibull breakdown-strength parameters of XLPE at different aging times in different solutions. (**a**) Scale parameter *α*; (**b**) Shape parameter *β*.

To provide a clearer visualization of the statistical results, Figure 9 illustrates the variations of Weibull scale parameter (*α*) and shape parameter (*β*) with aging time in different solutions. The figure highlights that seawater and NaCl environments lead to the most pronounced reductions in α and β, while Na_2_SO_4_ and NaHCO_3_ solutions exhibit relatively milder effects.

### 3.2. Material Structure Variation Analysis

#### 3.2.1. Chemical Structure Evolution (FTIR Analysis)

**Figure 10:**
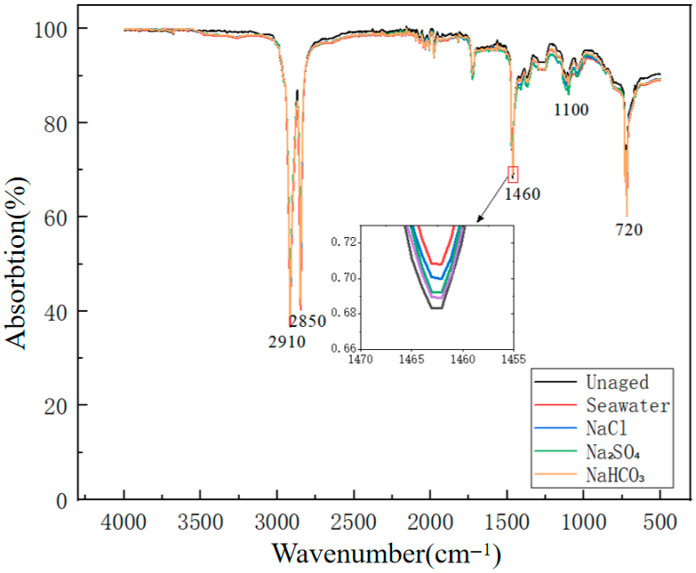
Comparative FTIR spectra of XLPE specimens before and after 672 h aging in different corrosive solutions.

Fourier Transform Infrared Spectroscopy (FTIR) serves as an effective method for analyzing molecular structural changes in polymers, enabling quantitative characterization of chemical bond cleavage and functional group evolution patterns in XLPE under different anionic degradation environments. Figure 10 presents comparative FTIR spectral analysis results for unaged XLPE specimens and specimens corroded for 672 h in different solutions.

From the spectral characteristics in Figure 10, typical methylene (–CH_2_–) characteristic absorption peaks were observed in all specimens at 2910 cm^−1^, 2850 cm^−1^, 1460 cm^−1^, and 720 cm^−1^, corresponding, respectively, to asymmetric stretching vibration, symmetric stretching vibration, scissoring deformation vibration, and in-plane rocking vibration of methylene groups. Intensity variations of these characteristic peaks directly reflect the integrity degree of XLPE molecular main chains. Notably, after 672 h of aging, all specimens exhibited varying degrees of intensity attenuation in methylene characteristic peaks, with attenuation extent depending on the corrosive medium, where seawater and NaCl solutions showed the most significant effects.

To quantitatively analyze the influence of different degradation conditions on XLPE molecular chain structure, this study employed the methylene index method for analysis [33]. This method effectively eliminates the impact of specimen thickness variations on test results by calculating the ratio of absorption peak area at specific wavenumbers to the internal standard peak area. The calculation formula for methylene index *R* is as follows:

(3) $$R=\frac{A_{x}}{A_{2010}}$$

where $A_{x}$ represents the absorption peak area at the wavenumber to be analyzed, and $A_{2010}$ represents the area of the internal standard peak at 2010 cm^−1^. The selection of 2010 cm^−1^ as the internal standard peak is based on it being a combination frequency vibration peak of 1303 cm^−1^ (non-crystalline structure absorption peak) and 720 cm^−1^ (crystallization-sensitive peak), which remains relatively stable during degradation processes and meets the basic requirements for an internal standard peak.

The methylene index variations for each characteristic peak calculated using Equation (3) are shown in Table 3. The data demonstrate that after 672 h of aging, all characteristic peaks exhibited significant decreases in methylene indices, with the magnitude of decrease clearly dependent on the type of corrosive medium. Specifically, at 2910 cm^−1^ and 2850 cm^−1^, seawater solution caused the most severe decreases in methylene indices, declining from 487.32 and 462.17 in the unaged state to 356.85 and 334.29 after aging, representing decreases of 26.8% and 27.7%, respectively. NaCl solution showed the second most significant impact, while Na_2_SO_4_ and NaHCO_3_ solutions had relatively smaller effects.

**Figure 11:**
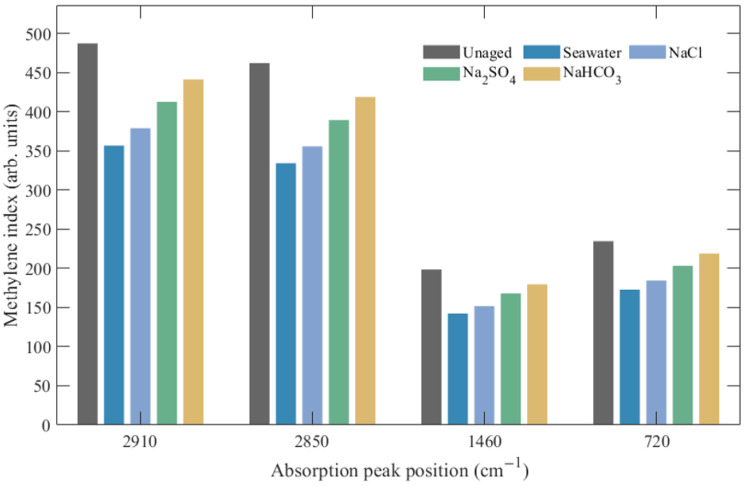
Variations of XLPE methylene indices at different absorption peaks under various degradation conditions.

To complement the numerical data in Table 3, Figure 11 presents the variations of methylene indices at the four characteristic absorption peaks in bar chart form. The visualization highlights that seawater and NaCl solutions cause the most pronounced decreases across all peaks, while Na_2_SO_4_ and NaHCO_3_ solutions exhibit comparatively smaller reductions.

The data in Table 3 indicate that all characteristic peaks showed significant decreases in methylene indices, with the magnitude of decrease clearly dependent on the type of corrosive medium. The change at 1460 cm^−1^ for methylene scissoring vibration was most pronounced, with the methylene index in seawater solution decreasing from 198.47 to 142.33, representing a decrease of 28.3%. This wavelength change directly reflects the degree of polymer main chain cleavage, indicating that the synergistic action of multiple anions in seawater significantly accelerated XLPE molecular chain degradation processes. NaCl solution showed a decrease of 23.5%, while Na_2_SO_4_ and NaHCO_3_ solutions showed decreases of 15.4% and 9.6%, respectively, presenting a clear stepped decreasing trend. The rocking vibration peak at 720 cm^−1^ is closely related to XLPE crystallinity, with the index decrease in seawater solution reaching 26.3%, reflecting molecular chain cleavage accompanied by crystalline structure destruction.

The action mechanisms of different anions on XLPE show significant differences. Cl^−^ ions, as strong nucleophiles, directly attack C–H bonds and initiate free radical chain reactions [34], leading to rapid molecular chain cleavage. SO_4_^2−^ ions primarily destroy molecular structure through oxidation reactions, while HCO_3_^−^ ions promote hydrolysis reactions by altering local pH values. The synergistic effects of multiple anions in seawater exhibit non-linear superposition characteristics, with their damage to molecular chains exceeding that of any single ion, originating from mutual promoting effects between different ions and complex electrochemical reaction processes. Spectral analysis results showed that while no obvious new peaks were observed in the carbonyl region of 1700-1750 cm^−1^, enhanced hydroxyl stretching vibration was detected in the 3200-3600 cm^−1^ range, related to increased material hygroscopicity and surface hydrogen bonding interactions. FTIR analysis revealed the differential impact mechanisms of various anions on XLPE chemical structure, with systematic decreases in methylene indices quantitatively characterizing molecular chain cleavage degrees, providing molecular-level evidence for establishing degradation-performance correlation models.

#### 3.2.2. Microscopic Morphology Changes (SEM Analysis)

**Figure 12:**
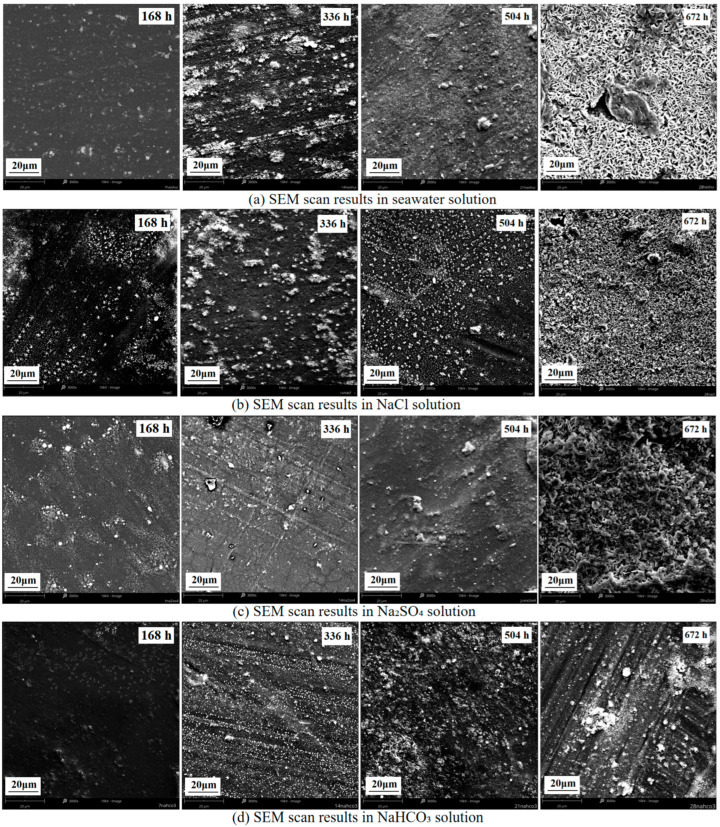
Surface morphology change patterns of XLPE specimens after aging in different corrosive solutions. (**a**) Seawater solution; (**b**) NaCl solution; (**c**) Na_2_SO_4_ solution; (**d**) NaHCO_3_ solution.

Scanning Electron Microscopy (SEM) was employed to reveal XLPE surface degradation evolution processes and microstructural change patterns under different anionic environments. Figure 12 presents surface morphology changes of XLPE specimens in four corrosive solutions after 168 h, 336 h, 504 h, and 672 h aging.

SEM analysis results demonstrate that XLPE surface morphology under all corrosive environments underwent similar evolutionary stages: initial appearance of scattered white deposits, peak deposit density in the intermediate stage followed by reduction, and significant surface roughness increase in the later stage accompanied by crack network formation. However, the evolution rate and final morphological characteristics differed significantly depending on the corrosive medium. After 672 h in seawater environment, surfaces showed the most severe crack networks and pore structures, NaCl solution exhibited similar but somewhat milder degradation characteristics, Na_2_SO_4_ solution formed networked degradation morphology, while NaHCO_3_ solution showed the least surface damage.

The appearance and disappearance processes of surface white deposits reflect degradation reaction characteristics involving different anions. In Cl^−^-containing environments, initial Cl^−^ attack on C–H bonds generates chlorinated alkane intermediates, which subsequently oxidize to form carbonyl compounds and ultimately decompose, causing dynamic changes in deposits. SO_4_^2−^ ions generate sulfate ester intermediates through oxidation reactions, gradually destroying polymer chain structure and forming characteristic networked degradation morphology. HCO_3_^−^ ions primarily promote hydrolysis reactions through pH adjustment, with relatively slow degradation progression. The temporal evolution of surface morphology reveals staged characteristics of the degradation process through initial nucleation, intermediate expansion, and later interconnection stages, corresponding to gradual XLPE molecular chain degradation: local chemical bond cleavage forms microscopic defects, defect expansion generates degradation products, and ultimate interconnection forms macroscopic crack networks. The multi-ion synergistic action in seawater significantly accelerated this evolution process, making surface damage exceed that in any single-ion environment.

Quantitative changes in surface roughness showed good correlation with macroscopic electrical performance degradation results. The most severe surface damage in seawater environment corresponded to the most significant volume resistivity decrease and breakdown field strength degradation, verifying the intrinsic relationship between microstructural changes and macroscopic performance deterioration. SEM analysis elucidated the differences in degradation mechanisms of various anions from a microscopic morphological perspective, providing important morphological evidence for establishing structure-performance correlation models.

### 3.3. Correlation Between Electrical Degradation and Microstructural Changes

The observed deterioration in electrical performance of XLPE is strongly reflected in the changes of its molecular and morphological structure. The reduction in volume resistivity and breakdown strength is accompanied by a marked decrease in the methylene index detected by FTIR, indicating the occurrence of molecular chain scission and oxidation reactions. The rise in dielectric constant and loss factor corresponds to the generation of polar functional groups, which alter charge transport and increase dielectric losses. SEM images provide direct evidence of surface damage, where the formation of cracks, voids, and roughened regions coincide with the decline of electrical integrity.

Seawater-aged specimens exhibit the most severe electrical degradation, which is consistent with the lowest methylene indices and the most extensive crack networks observed by SEM. NaCl and Na_2_SO_4_ solutions cause substantial but less pronounced deterioration, in line with moderate FTIR shifts and surface damage patterns. The weakest changes appear in NaHCO_3_ solution, where both electrical and microstructural degradation remain limited.

These correlations demonstrate that electrical aging behavior cannot be separated from underlying structural transformations. The severity of resistivity loss, dielectric instability, and breakdown reduction mirrors the extent of chemical bond scission and surface defects. The more drastic deterioration in seawater compared with single-ion solutions supports the view that nonlinear synergistic effects are at play, where multiple ions accelerate degradation beyond the sum of their individual contributions.

## 4. Degradation Mechanism Discussion

To situate our interpretations within the state of the art, we note that moisture-assisted degradation of XLPE in marine or saline environments typically initiates from water-tree precursors at the semiconductive screen/insulation interface and evolves with temperature-dependent kinetics; these processes coexist with oxidation-driven chain scission evidenced by FTIR indices and with space-charge-induced field distortion under electrical stress. Accordingly, the mechanistic picture discussed below is consistent with recent reports on vented water-tree inception in wet-aged HV XLPE, temperature-controlled water-tree propagation [35,36].

### 4.1. Single Anion Degradation Mechanism

Based on the experimental results presented above, different anions exhibit fundamentally distinct degradation mechanisms toward XLPE, displaying clear patterns in their pathways and intensities of action.

The chloride ion (Cl^−^) degradation mechanism demonstrates the strongest corrosive effect, primarily through nucleophilic substitution reactions that directly attack the C–H bonds in XLPE molecular chains. Under 90 °C high-temperature conditions, Cl^−^ ions first undergo nucleophilic substitution reactions with the polymer backbone:

(4) $$-{\mathrm{CH}}_{2}-{\mathrm{CH}}_{2}-+{Cl}^{-}\to -{\mathrm{CH}}_{2}-\mathrm{CHCl}-+H^{+}+e^{-}$$

The chloroalkane intermediate products generated by this reaction further undergo β-elimination reactions at high temperatures, forming unsaturated bonds and releasing HCl. The generated unsaturated bonds make the molecular chains more susceptible to oxidative attack, initiating free radical chain reactions:

(5) $$-CH=CH-+O_{2}\to -\mathrm{CH}(\mathrm{OO}•)-\mathrm{CH}-$$

The formation of peroxy radicals leads to molecular chain scission and the generation of carbonyl compounds. This process explains the significant decrease in methylene indices observed in FTIR analysis and the white deposit phenomena observed in SEM.

The sulfate ion (SO_4_^2−^) degradation mechanism primarily destroys XLPE structure through oxidation reactions. SO_4_^2−^ ions act as strong oxidizing agents, reacting with polymer molecular chains under high-temperature conditions:

(6) $$-{\mathrm{CH}}_{2}-{\mathrm{CH}}_{2}-+{SO}_{4}^{2-}+H^{+}\to {-{\mathrm{CH}}_{2}-\mathrm{CH}(\mathrm{OSO}}_{3}H)-$$

The formed sulfate ester intermediates undergo hydrolysis in the presence of water, generating hydroxyl groups that further oxidize to form carbonyl compounds, resulting in molecular chain structure destruction. The network degradation morphology observed in SEM analysis precisely represents the spatial manifestation of this progressive oxidation reaction.

The bicarbonate ion (HCO_3_^−^) degradation mechanism is relatively mild, primarily promoting hydrolysis reactions by adjusting local pH values. Under 90 °C conditions, HCO_3_^−^ undergoes thermal decomposition to produce OH^−^ ions, increasing local pH values and promoting alkaline hydrolysis of XLPE molecular chains. The degradation process under this mechanism is relatively slow, consistent with the minimal performance degradation observed experimentally.

The order of single anion degradation strength (Cl^−^ > SO_4_^2−^ > HCO_3_^−^) is directly related to their chemical activities and reaction mechanisms: the strong nucleophilic nature of Cl^−^ enables direct C–H bond cleavage, and the oxidizing properties of SO_4_^2−^ cause progressive molecular chain degradation, while HCO_3_^−^ action is mainly limited to pH adjustment-induced indirect hydrolysis.

### 4.2. Multi-Ion Synergistic Action Mechanisms

The coexistence of multiple anions (Cl^−^, SO_4_^2−^, HCO_3_^−^) in seawater environments produces significant synergistic degradation effects, with damage to XLPE exceeding that of any single ion environment. This non-linear superposition characteristic stems from mutual promotion between ions and complex electrochemical reaction processes. The synergistic oxidative chain reaction mechanism is the core of multi-ion synergistic action. Cl^−^ ions first create active sites on XLPE molecular chains through nucleophilic substitution reactions, followed by SO_4_^2−^ ions undergoing oxidation reactions at these sites, accelerating molecular chain scission:

(7) $$-CHCl-+{SO}_{4}^{2-}\to -C\left(O\right)-+{Cl}^{-}+{SO}_{3}^{2-}+H^{+}$$

The generated SO_3_^2−^ further reacts with water to produce sulfurous acid, lowering local pH values. The acidic environment promotes HCO_3_^−^ decomposition, producing more active OH^−^ ions to participate in hydrolysis reactions. This cyclic reaction mechanism significantly improves overall degradation efficiency.

Electrochemical coupling effects further amplify the synergistic action. Different ions form microcell structures on XLPE surfaces, promoting charge transfer and the progression of redox reactions. Simultaneously, polymer molecular chains undergo oxidation reactions at the anode:

(8) $$-{\mathrm{CH}}_{2}-{\mathrm{CH}}_{2}-\to {-{\mathrm{CH}}_{2}-CH}^{+}-+H^{+}+2e^{-}$$

This electrochemical process accelerates molecular chain scission and degradation product formation.

Ion transport synergistic mechanisms also constitute important pathways for synergistic action. Similar findings were reported for ionic/wet aging of XLPE, where combined electrical, thermal, and mechanical evaluations confirmed accelerated moisture uptake and deterioration of dielectric properties [37]. The presence of different ions alters solution ionic strength and conductivity, promoting ion diffusion and penetration within XLPE micropore structures. The small ionic radius of Cl^−^ ions enables them to penetrate material interiors first, followed by larger SO_4_^2−^ ions entering along channels opened by Cl^−^.

The macroscopic manifestation of multi-ion synergistic action is reflected in various aspects of experimental results: the 28.3% methylene index decrease in seawater environments exceeds the maximum value in single ion environments (23.5%), while the 93% volume resistivity decrease and 23.2% breakdown strength degradation are significantly higher than in single ion environments. The most severe crack networks and pore structures observed in SEM analysis further confirm the destructive nature of multi-ion synergistic degradation.

To further substantiate the existence of nonlinear superposition effects, it is worth highlighting how FTIR and SEM results directly support this interpretation. FTIR spectra revealed that the decrease in methylene index at 1460 cm^−1^ reached 28.3% in seawater, which is significantly higher than the decreases observed in any single-ion environment (maximum 23.5% for Cl^−^). This excess degradation indicates that the combined action of multiple anions promotes molecular chain scission beyond the additive effects of individual ions. In parallel, SEM observations showed that surface crack networks and pore structures in specimens aged in seawater were far more extensive than in NaCl, SO_4_^2−^, or HCO_3_^−^ solutions alone. The severity of morphological deterioration in seawater is thus consistent with accelerated structural breakdown caused by coupled oxidative, hydrolytic, and electrochemical processes. Together, the quantitative FTIR indices and qualitative SEM evidence confirm that the degradation pathways in seawater cannot be explained by linear addition of single-ion effects, but instead arise from nonlinear synergistic interactions among Cl^−^, SO_4_^2−^, and HCO_3_^−^.

## 5. Conclusions

This study provides the first systematic comparison of the differential degradation mechanisms of major anions in seawater (Cl^−^, SO_4_^2−^, HCO_3_^−^) on XLPE insulation materials and reveals the non-linear superposition characteristics of multi-ion synergistic action. Through 672-h high-temperature accelerated aging tests combined with multi-scale characterization analysis, a complete correlation system from microscopic molecular structure to macroscopic electrical performance was established. The conclusions obtained are as follows:

1. Seawater environments cause the most severe XLPE performance degradation, with volume resistivity decreasing by 93% and breakdown strength declining from 75.37 kV/mm to 57.84 kV/mm (23.2% reduction), significantly exceeding the impact of any single ion environment.
2. Microscopic structural analysis confirmed the intrinsic mechanisms underlying macroscopic performance changes—seawater environments resulted in up to 28.3% reduction in the methylene index at 1460 cm^−1^ and formation of the most severe crack networks and pore structures on surfaces.
3. Single anion degradation strength follows the pattern Cl^−^ > SO_4_^2−^ > HCO_3_^−^, directly related to their chemical activities and reaction mechanisms.

Degradation mechanism analysis reveals that Cl^−^ ions demonstrate the strongest corrosive effect through nucleophilic substitution reactions directly attacking C–H bonds, SO_4_^2−^ ions primarily destroy molecular chain structures through oxidation reactions, and HCO_3_^−^ ions promote hydrolysis reactions through pH adjustment. Multiple anions in seawater produce non-linear superposition synergistic degradation through coordinated oxidative chain reactions and electrochemical coupling effects, with damage exceeding that of any single ion environment.

This study establishes that multi-ion synergistic degradation in marine environments is the key factor causing XLPE submarine cable insulation performance degradation. The research findings provide important theoretical foundations and technical support for optimization design of submarine cable insulation materials, protective strategy formulation, and service life prediction, holding significant engineering value for ensuring long-term reliability of marine energy transmission systems.

## Figures and Tables

**Table 1:** Chemical reagent preparation scheme for four corrosive solutions.

Solution Name	Main Chemical Reagent	Dosage (mg/L)
NaCl Solution	NaCl	3.048 × 10^4^
Na_2_SO_4_ Solution	Na_2_SO_4_	3.92 × 10^3^
NaHCO_3_ Solution	NaHCO_3_	2.0 × 10^2^
Seawater Solution	NaCl	3.048 × 10^4^
	Na_2_SO_4_	3.92 × 10^3^
	NaHCO_3_	2.0 × 10^2^

**Table 2:** Weibull distribution parameters for XLPE breakdown strength at different thermal aging times.

Solution	Aging Time (h)	*α *(kV/mm)	*β*
-	0	75.37	45.0
Seawater	168	68.62	33.2
Seawater	336	64.37	27.6
Seawater	504	61.12	24.8
Seawater	672	57.84	20.9
NaCl	168	69.28	35.4
NaCl	336	65.31	30.6
NaCl	504	61.54	27.2
NaCl	672	57.90	22.7
Na_2_SO_4_	168	70.14	39.5
Na_2_SO_4_	336	67.38	35.6
Na_2_SO_4_	504	64.23	30.8
Na_2_SO_4_	672	60.65	26.3
NaHCO_3_	4.0	71.20	39.0
NaHCO_3_	1.5	68.40	34.0
NaHCO_3_	6.0	66.10	30.5
NaHCO_3_	672	64.00	27.5

**Table 3:** Changes in XLPE methylene indices under different degradation conditions.

Absorption Peak Position/cm^−1^	Unaged	Seawater Solution	NaCl Solution	Na_2_SO_4_ Solution	NaHCO_3_ Solution
2910	487.32	356.85	378.94	412.67	441.28
2850	462.17	334.29	355.82	389.45	418.93
1460	198.47	142.33	151.76	167.89	179.52
720	234.61	172.84	184.37	203.28	218.95

## Data Availability

The data presented in this study are available on request from the corresponding author.
